# Ovarian Anti-Müllerian Hormone Expression in Hyperprolific Sows: Associations with Reproductive Stage and Parity, with Elevated Expression in the Corpus Luteum During the Luteal Stage

**DOI:** 10.3390/ani16192974

**Published:** 2026-09-22

**Authors:** Duangkamol Lewchalermwong, Yosua Kristian Adi, Preechaphon Taechamaeteekul, Em-on Olanratmanee, Sawang Kesdangsakonwut, Padet Tummaruk

**Affiliations:** 1 Department of Veterinary Nursing, Faculty of Veterinary Technology, Kasetsart University, Bangkok 10900, Thailand; cvtdmp@ku.ac.th; 2 Department of Reproduction and Obstetrics, Faculty of Veterinary Medicine, Universitas Gadjah Mada, Yogyakarta 55281, Indonesia; yosua.kristian.a@ugm.ac.id; 3 Centre of Excellence in Swine Reproduction, Department of Obstetrics, Gynaecology and Reproduction, Faculty of Veterinary Science, Chulalongkorn University, Bangkok 10330, Thailand; taechamaeteekul.p@gmail.com; 4 Faculty of Veterinary Medicine, Rajamangala University of Technology Tawan-ok, Chonburi 20110, Thailand; em-on_ol@rmutto.ac.th; 5 Department of Veterinary Pathology, Faculty of Veterinary Science, Chulalongkorn University, Bangkok 10330, Thailand; sawang.k@chula.ac.th

**Keywords:** anti-müllerian hormone, follicle, ovary, parity, prolific sows

## Abstract

Although anti-Müllerian hormone (AMH) is widely recognized as a marker of ovarian reserve, its expression in ovarian tissue of hyperprolific sows remains poorly characterized. This study investigated ovarian AMH expression across reproductive stages and evaluated its associations with ovulation rate and parity in hyperprolific Landrace × Yorkshire sows. Ovarian tissues from thirty-one sows were collected after slaughter and classified according to reproductive stage, parity, and ovulation rate. AMH expression was assessed in primordial, primary, secondary, and antral follicles, as well as corpora lutea, using immunohistochemistry and was quantified with the H-score method. Anti-Müllerian hormone was localized to granulosa cells and oocytes throughout follicular development, as well as to luteal cells in the corpus luteum. Expression within ovarian follicles did not differ among follicle types, reproductive stages, parity groups, or ovulation rate classes. In contrast, luteal cells exhibited significantly greater AMH expression during the luteal phase than during the follicular phase (*p* < 0.001). Antral follicles in primiparous sows appeared to show higher expression than those in multiparous sows, whereas no association was observed between AMH expression and ovulation rate. These findings demonstrate consistent ovarian AMH expression throughout follicular development and identify stage-dependent expression in luteal tissue. Notably, AMH expression was not associated with ovulation rate, indicating that AMH may reflect the size of the growing follicle pool rather than ovulatory capacity. The age-related trend observed in antral follicles supports the potential value of AMH as an indicator of reproductive longevity in hyperprolific sows.

## Simple Summary

Better tools are needed to identify animals with healthy ovaries so that farmers can make informed breeding and management decisions. This study examined the distribution of an anti-Müllerian hormone that reflects ovarian activity in the ovaries of highly productive sows and evaluated whether its expression was associated with reproductive stage, the number of eggs released during ovulation, and the number of previous pregnancies. The hormone was detected throughout follicle development. Its expression was higher in this tissue during the active luteal stage of the reproductive cycle, while younger sows appeared to exhibit higher expression in developing follicles than older sows. However, hormone expression was not related to the number of eggs released during a reproductive cycle. These findings improve our understanding of ovarian function in highly productive sows and indicate that this hormone may serve as a useful indicator of ovarian reserve and long-term reproductive potential. This knowledge may help improve breeding decisions, reproductive management, animal welfare, and the sustainability of commercial pig production.

## 1. Introduction

Anti-Müllerian hormone (AMH), also known as Müllerian-inhibiting substance, is a glycoprotein belonging to the transforming growth factor-β (TGF-β) superfamily [1,2,3]. In females, AMH is secreted by the granulosa cells of growing ovarian follicles and plays an important role in regulating folliculogenesis [4]. It inhibits the initial recruitment of primordial follicles and modulates follicular sensitivity to follicle-stimulating hormone (FSH), thereby contributing to the maintenance of the ovarian reserve [5,6]. In pigs, AMH expression is first detected in primary follicles, reaches its peak levels in preantral and small antral follicles, and subsequently declines in larger, FSH-dependent follicles [2]. In pubertal gilts, strong AMH immunolocalization has been observed in granulosa cells of healthy follicles at several developmental stages, including preantral, early antral, large antral, and preovulatory follicles [7]. By contrast, gilts culled because of anestrus exhibit reduced ovarian AMH immunoexpression, which may be associated with lower circulating AMH concentrations [7]. These findings suggest that impaired AMH function during follicular development may contribute to anestrus in replacement gilts [7].

Circulating AMH concentrations are positively correlated with the number of antral follicles, supporting the potential use of AMH as a biomarker of ovarian reserve and reproductive capacity [8]. Recent evidence also indicates that AMH concentrations may predict important reproductive traits, including age at puberty, ovulation rate, superovulatory response, and lifetime reproductive performance in gilts and sows [9,10]. However, the distribution and localization of AMH within ovarian tissue, particularly in relation to ovulation rate and parity, remain poorly characterized.

Modern hyperprolific sow lines are characterized by high ovulation rates and large litter sizes, which impose substantial physiological demands on ovarian function and follicular development [11,12,13]. The mean ovulation rate in hyperprolific sows is approximately 26.5, with considerable individual variation ranging from 14 to 40 ovulations [14]. Under commercial tropical conditions, up to 24.5% of hyperprolific sows have been reported to produce more than 20 total piglets per litter [13]. Moreover, each additional piglet born in the first litter is associated with an increase of 0.35 piglets in the subsequent litter [13]. Nevertheless, excessively large first-parity litters have been associated with an increased risk of second-parity syndrome and reduced sow longevity, indicating a potential trade-off between early reproductive output and lifetime productivity [13]. These findings highlight the importance of appropriate gilt selection and lactation management to sustain reproductive efficiency, particularly under tropical production conditions [11].

The physiological demands associated with hyperprolificacy provide an important context for interpreting ovarian AMH expression. Repeated reproductive cycles, large litters, and substantial lactational demands may place considerable pressure on maternal resource allocation, potentially contributing to accelerated reproductive aging [11,12,13]. Age- and reproductive-stage-related patterns of AMH expression may therefore offer insights into ovarian changes accompanying sustained reproductive demands, although they do not establish accelerated aging or predict reproductive longevity. Tropical conditions are particularly relevant because high temperatures and humidity can impose additional thermal stress, potentially limiting feed intake and recovery between reproductive cycles [11]. Examining AMH expression in hyperprolific sows under these conditions provides a basis for investigating how reproductive demands and environmental stress may jointly influence ovarian physiology.

Reproductive aging is characterized by a progressive decline in follicular reserve and ovarian function and represents a major constraint to maintaining sow productivity throughout life. AMH concentrations decline with age in several mammalian species [15,16,17,18], including humans [19,20,21,22,23], and may therefore provide valuable insight into reproductive senescence and the sustainability of fertility in sows. This issue is particularly relevant in hyperprolific sow lines, in which high ovulation rates and large litter sizes may accelerate follicular depletion or compromise oocyte quality over successive parities. Despite growing interest in AMH as a biomarker of ovarian reserve, information regarding its spatial expression within porcine ovarian tissue across reproductive stages and parities remains limited, especially in hyperprolific sows maintained under commercial conditions. Furthermore, the relationships between ovarian AMH expression and key reproductive characteristics, including ovulation rate and parity, have not been fully elucidated.

A better understanding of these relationships may clarify the biological mechanisms underlying variation in sow reproductive performance and support the development of improved strategies for gilt selection and sow retention. Therefore, the objectives of the present study were to (*i*) characterize AMH expression in the ovarian tissues of hyperprolific sows according to reproductive stages, and (*ii*) evaluate AMH expression associations with ovulation rate and parity. We hypothesized that ovarian AMH expression would vary among follicular developmental stages and parity groups and would be associated with ovulation rate, reflecting differences in ovarian functional capacity among hyperprolific sows.

## 2. Materials and Methods

### 2.1. Animals

The study was approved by the Institutional Animal Care and Use Committee (IACUC) of Chulalongkorn University (Approval No. 2331095) and was conducted in accordance with the university’s ethical guidelines for the use of experimental animals. Reproductive tracts were collected from 31 hyperprolific Landrace × Yorkshire crossbred sows, characterized by a high average total number of piglets born per litter (more than 16 piglets/litter) and categorized into three parity groups: parities 1 (*n* = 10), 2–5 (*n* = 12), and 6–9 (*n* = 9). Parity number, birth date, and body weight at culling were obtained from herd records using individual identification numbers. All sows were culled for management-related reasons and showed no clinical signs of reproductive disorders. Slaughter was performed at a commercial abattoir following standard procedures. The reproductive history of each sow, including the number of piglets born in each previous parity, was obtained from the individual sow identification records before transportation to the slaughterhouse. During the slaughter process, the collected reproductive organs were identified using the sow identification number and matched with the corresponding reproductive records. The average number of piglets born was subsequently calculated for each experimental group.

Immediately after slaughter, the entire reproductive tract, including the ovaries (Figure 1), oviducts, uterus, vagina, vestibule, and vulva, was placed on ice and transported to the laboratory for examination within 12 h of collection. A gross morphological assessment was conducted, and the ovaries were separated from the mesovarium and classified as being in either the follicular or luteal phase based on established morphological criteria. Briefly, the luteal phase was defined by the presence of corpora hemorrhagica or corpora lutea, whereas the follicular phase was defined by the presence of 7–12 mm follicles with or without corpora albicantia (Figure 1). The diameter of follicles was measured using a vernier caliper. The numbers of follicles and corpora lutea were recorded for each ovary, and the total ovulation number was calculated as the sum of corpora lutea from both ovaries. Ovulation rate was categorized into three groups based on the frequency distribution of the observed number of ovulations, yielding groups of comparable size: low (10–22 ovulations, *n* = 8), moderate (23–29 ovulations, *n* = 11), and high (30–40 ovulations, *n* = 7). Ovulation data were available for 26 sows, as corpora lutea could not be identified in the remaining five sows classified as being in the follicular phase, as sows in the follicular phase have not yet ovulated and lack of corpora lutea.

### 2.2. Tissue Processing and Immunohistochemistry

Ovarian tissue samples were fixed in 4.0% paraformaldehyde for 48 h, processed using an automated tissue processor (Tissue-Tek VIP 5 Jr., Sakura, Tokyo, Japan), and embedded in paraffin blocks (Tissue-Tek TEC, Sakura, Tokyo, Japan). Paraffin-embedded tissues were sectioned into 5-µm sections using a microtome (Shandon, Anglia Scientific Instrument Ltd., Cambridge, UK). Sections were deparaffinized in xylene and rehydrated through a graded ethanol series. Antigen retrieval was performed by heating sections in 10 mM citrate buffer (pH 6.0) in a microwave at 500 W for 10 min. Endogenous peroxidase activity was blocked with freshly prepared 3.0% hydrogen peroxide (H2O2) at room temperature (RT) for 15 min. To reduce non-specific binding, sections were incubated with 2.0% normal horse serum (Vector Laboratories Inc., Burlingame, CA, USA) at RT for 30 min. AMH immunoreactivity was assessed using a mouse monoclonal antibody (sc-365643, Santa Cruz Biotechnology, Inc., Dallas, TX, USA) at a dilution of 1:200, incubated overnight at 4 °C. Antibody specificity was confirmed using negative controls. For negative controls, the primary antibody was replaced with phosphate-buffered saline, which showed no positive staining. Sections were then incubated with the DAKO EnVision reagent kit (K4063, Agilent Technologies, Santa Clara, CA, USA) at RT for 15 min. Signal visualization was performed using 3,3′-diaminobenzidine (ImmPACT™ DAB substrate, Vector Laboratories Inc., Burlingame, CA, USA) at RT for 2 min. This incubation time was applied to all sections. Sections were counterstained with Mayer’s hematoxylin and mounted using Permount^®^ mounting medium (Fisher Scientific, Pittsburgh, PA, USA).

### 2.3. Immunohistochemical Analysis and Scoring

Images of immunohistochemically stained ovarian tissues were captured using a digital slide scanner (Pannoramic SCAN, 3DHistech Co., Ltd., Budapest, Hungary) and analyzed with image analysis software (CellQuant module version 2.3, 3DHistech Co., Ltd., Budapest, Hungary). Ovarian follicles were classified into primordial, primary, secondary, and antral stages based on established criteria [24]. Briefly, primordial follicles were defined as those containing an oocyte surrounded by a single layer of flattened pre-granulosa cells, without cuboidal granulosa cells. Primary follicles were defined as those containing a centrally located oocyte surrounded by either a mixture of flattened pre-granulosa cells and cuboidal granulosa cells or by a single layer of cuboidal granulosa cells. Secondary follicles were defined as those exhibiting a visible zona pellucida and/or two or more layers of granulosa cells, without a visible antrum. Antral follicles were defined as those exhibiting a visible zona pellucida and/or multiple layers of granulosa cells, with a visible antral cavity. A histological slide per ovary per sow was randomly selected for analysis, and AMH immunostaining was evaluated in every follicle present on that slide. The H-score values obtained were then averaged to generate a mean value for each follicle type. The intensity of AMH expression at each follicular stage was quantified using the H-score method, calculated as follows: H-score = (1 × % weakly stained cells) + (2 × % moderately stained cells) + (3 × % strongly stained cells) [25]. A total of 25 fields of view (each 8 × 10^4^ µm^2^) were analyzed for DAB staining intensity, which was categorized as negative, weak, moderate, or strong. H-scores for regions of interest were expressed on a scale of 0–300. All H-score assessments were performed by a single researcher to minimize inter-observer variability.

### 2.4. Statistical Analysis

All statistical analyses were performed using SAS software version 9.4 (SAS Institute Inc., Cary, NC, USA). Descriptive statistics for continuous variables, including sow age at culling, body weight, parity number, total number of piglets born per litter, and ovulation rate, were generated using the MEANS procedure. Continuous data related to reproductive performance and gross reproductive morphology were analyzed using the General Linear Model (GLM) procedure. Fixed effects included parity group (1, 2–5, and 6–9) and reproductive cycle stage (follicular and luteal). The interaction term was initially tested but excluded from the final model due to lack of significance (*p* > 0.20).

The H-score of AMH expression in each follicle type and in corpora lutea was analyzed using the GLM procedure, with parity group, reproductive cycle stage, and their two-way interaction included as fixed effects. The interaction term was removed from the final model due to non-significance (*p* > 0.20). Additionally, the effect of ovulation rate class on AMH expression was analyzed using the GLM procedure, with parity group and ovulation class (low, moderate, high) included as fixed effects. Parity group was used as a proxy for reproductive age, as age at culling and parity were strongly correlated in this population; age was therefore not included as a continuous covariate in the same model. The interaction between these factors was also tested but excluded due to non-significance (*p* > 0.20). Reproductive cycle stage was not included in this model, as ovulation data were primarily obtained from the luteal phase. The normality of residuals for the dependent variable (H-score) was assessed using the Shapiro–Wilk test. As the data were not normally distributed, variables were log-transformed and reanalyzed using the same statistical models. The mean H-scores of AMH expression among different follicle types and corpora lutea were compared using the GLM procedure, with follicular stage (primordial, primary, secondary, antral follicles, and corpora lutea) included as a fixed effect. For all models, least-squares means and their standard errors (SEM) were calculated, and differences among groups were evaluated using the Tukey–Kramer adjustment for multiple comparisons. Statistical significance was defined as *p* < 0.05.

## 3. Results

### 3.1. Descriptive Statistics

Descriptive statistics of sow characteristics and reproductive performance parameters are presented in Table 1. At culling, sows had a mean age of 787.5 ± 325.6 days and a mean body weight of 215.7 ± 29.1 kg. Moreover, the mean age and body weight for each parity group are presented in Table 1. The average parity at culling was 3.3 ± 2.3 (range: 1–9). Regarding reproductive performance, the mean ovulation rate, assessed in 26 sows, was 25.5 ± 7.9. Based on ovulation number, sows were classified into three categories: low (10–22; 30.8%), moderate (23–29; 42.3%), and high (30–40; 26.9%). The mean total number of piglets born per litter increased with parity, ranging from 15.9 ± 2.5 in primiparous sows to 17.9 ± 4.3 in sows of parity 6–9. Substantial inter-individual variation was observed, with litter sizes ranging from 3 to 31 piglets (Table 1).

### 3.2. Localization of AMH Immunoexpression in Porcine Ovary

AMH immunolocalization was observed across multiple stages of follicular development, including primordial (Figure 2A), primary (Figure 2B), secondary (Figure 2C), and antral follicles (Figure 2D). Immunoreactivity was localized to the cytoplasm of granulosa cells surrounding the oocyte and to the oocyte cytoplasm at all follicular stages (Figure 2E). No nuclear staining for AMH was detected in either granulosa cells or oocytes at any stage. The intensity of AMH staining varied among follicular stages, with mean H-scores ranging from 73.1 ± 62.9 in secondary follicles to 109.3 ± 73.3 in primary follicles. However, these differences were not statistically significant across follicle types or when compared with corpora lutea (Table 2). In addition, AMH immunoexpression was detected in the cytoplasm of luteal cells within the corpus luteum (Figure 2F).

### 3.3. Influence of Parity, Ovulation Rate, and Reproductive Stage on AMH Expression

Table 2 summarizes the AMH immunoexpression in ovarian follicles and the corpus luteum, stratified by parity, ovulation rate, and reproductive stage. Parity had no significant effect on AMH expression in any follicle type, including primordial (*p* = 0.687), primary (*p* = 0.520), secondary (*p* = 0.298), or antral follicles (*p* = 0.156), or in the corpus luteum (*p* = 0.853). AMH expression in antral follicles did not differ significantly between parity 1 sows and those of parities 2–5 (*p* = 0.089) or parities 6–9 (*p* = 0.092). Similarly, ovulation rate did not significantly influence AMH expression in primordial (*p* = 0.656), primary (*p* = 0.803), secondary (*p* = 0.504), or antral follicles (*p* = 0.749), or in the corpus luteum (*p* = 0.606). Reproductive stage (luteal vs. follicular) was also not associated with AMH expression in primordial (*p* = 0.529), primary (*p* = 0.994), secondary (*p* = 0.292), or antral follicles (*p* = 0.254). In contrast, AMH expression in the corpus luteum was significantly affected by reproductive stage, with higher H-scores observed during the luteal stage compared with the follicular stage (100.9 ± 10.8 vs. 24.7 ± 21.9, *p* < 0.001).

## 4. Discussion

AMH immunolocalization was consistently observed across multiple stages of follicular development, including primordial, primary, secondary, and antral follicles, with immunoreactivity confined to the cytoplasm of granulosa cells. This distribution is consistent with previous reports in pigs [2,10,16] and in other species [18,26,27] supporting granulosa cells as the principal site of AMH synthesis in the ovary. Beyond this established pattern, the present study also demonstrates reproductive-stage-dependent AMH expression in the corpus luteum, suggesting that AMH synthesis is not restricted to the follicular compartment. The presence of AMH in granulosa cells across all developmental stages suggests a role in the regulation of early folliculogenesis, likely through the inhibition of initial follicle recruitment and modulation of follicle-stimulating hormone (FSH) sensitivity in growing follicles [28]. In addition, AMH immunoexpression was detected in the cytoplasm of luteal cells, consistent with previous observations [2,29]. This finding suggests a potential involvement of AMH in luteal function or regression, although its specific role in luteal tissue remains to be elucidated. Notably, the present study distinguishes between active corpora lutea (luteal phase) and regressed corpora lutea (follicular phase), providing further insight into stage-specific AMH expression. Collectively, these findings indicate that AMH may contribute to the functional regulation of the corpus luteum in sows.

Quantitative analysis of AMH expression (H-score) revealed no significant differences among follicle types or between follicles and luteal cells, indicating relatively stable AMH expression throughout follicular development. In addition, AMH expression within follicles did not differ significantly between the follicular and luteal phases, consistent with previous reports suggesting that AMH production by granulosa cells is largely independent of gonadotropin fluctuations [26]. However, AMH expression in luteal cells was significantly higher during the luteal phase compared with the follicular phase, indicating that active corpora lutea may contribute more substantially to ovarian AMH production during this period. This observation may reflect the dynamic endocrine milieu following ovulation and highlights the need for further investigation into the role of AMH in luteal function.

AMH expression in antral follicles was numerically higher in primiparous than in multiparous sows, although the difference was not statistically significant. Thus, any association with reproductive age remains uncertain. Nevertheless, studies in pigs and other species have reported age-related declines in AMH associated with reduced follicular reserve and diminished follicular quality [2,27,29]. In sows, reproductive aging is characterized by impaired granulosa cell function, reduced oocyte competence, and increased follicular atresia [30]. These changes may contribute to decreased AMH expression and declining reproductive performance [11], providing a possible biological context for the numerical pattern observed in the present study. Supporting this, Fusapniran et al. [13] reported an increase in stillbirth incidence with advancing parity, from <6% in primiparous sows to >10% in sows with parity ≥9. Similarly, Chaiyapatmaetee et al. [31] observed a higher prevalence of intrauterine growth-restricted (IUGR) piglets among stillborn compared with liveborn piglets. These findings indicate that fetal growth restriction and stillbirth become more prevalent with advancing parity, reflecting reduced fetal viability and compromised ovarian function. Collectively, these observations support the potential utility of AMH as a biomarker for predicting reproductive longevity and productivity in sows. Even modest reductions in AMH expression with increasing parity may serve as early indicators of reproductive senescence. Further large-scale, parity-stratified longitudinal studies are warranted to validate the predictive value of AMH for identifying sows at risk of declining reproductive performance in commercial production systems.

No significant association was observed between ovulation number and AMH expression in any follicle type. This finding suggests that ovarian AMH expression may not directly reflect ovulatory output within a given estrous cycle, although the absence of a significant association should be interpreted cautiously. AMH is produced by granulosa cells in small- to medium-sized growing follicles, and its expression declines as follicles approach the preovulatory stage [2,19,20]. Ovarian AMH expression may therefore reflect the size and activity of the growing follicle population more closely than the number of ovulations in a particular cycle. This distinction is relevant to reproductive assessment [32]. Whereas ovulation rate reflects one component of reproductive potential within a given cycle, AMH may provide complementary information about follicular dynamics and ovarian function [19]. In commercial swine production, where early identification of gilts with high reproductive potential is important, circulating AMH has potential as a biomarker to support selection programs, although its ability to predict future reproductive performance requires further validation [2,7]. The immunohistochemical assessment used in the present study is impractical for routine field use. A practical herd-level application would instead require the measurement of circulating AMH concentrations. Further studies should examine the relationship between ovarian AMH expression and circulating AMH levels and determine whether serum AMH can provide reliable information about ovarian function and subsequent reproductive performance in commercial herds.

Reproductive stage (luteal vs. follicular phase) did not significantly affect AMH expression in ovarian follicles at any developmental stage, including primordial, primary, secondary, and antral follicles. This finding indicates that AMH expression within the follicular compartment remains relatively stable throughout the reproductive cycle, consistent with its established role in early folliculogenesis and the regulation of initial follicle recruitment [33,34,35]. In contrast, AMH expression in the corpus luteum was significantly influenced by reproductive stage, with markedly higher expression observed during the luteal phase than during the follicular phase, suggesting that luteal AMH expression may instead be linked to luteal cell differentiation rather than gonadotropin signaling. This pattern may reflect residual secretory activity of luteinized granulosa cells carried over from the preovulatory follicle, or new AMH synthesis by luteal cells, pointing to a previously unrecognized extrafollicular role [2]. The elevated H-scores during the luteal phase further support a potential regulatory role of AMH in luteal tissue; however, the physiological significance of this expression remains to be elucidated. These findings are consistent with the possibility that elevated luteal AMH may modulate FSH sensitivity, follicle recruitment, and luteal steroidogenesis, although the precise physiological significance of AMH in luteal tissue remains to be determined [19,36]. AMH may play a role in modulating luteal cell responsiveness to gonadotropins during luteolysis, although this remains speculative and warrants further investigation using markers of luteal cell differentiation.

A limitation of the present study is the relatively small sample size, and some parity and ovulation rate subgroups included a limited number of sows, which may have limited statistical power and the generalizability of the findings. Although a large number of follicles were examined, they were derived from 31 sows, so these findings should not be interpreted as fully independent observations. In addition, circulating AMH concentrations were not measured, precluding evaluation of their associations with ovarian immunohistochemical markers, follicular populations, and ovulation rate. Future studies involving larger populations and longitudinal measurements of circulating AMH are warranted to clarify these relationships and enhance the practical relevance of the findings for commercial production.

## 5. Conclusions

In conclusion, AMH expression was detected in both ovarian follicles and luteal cells. The principal novel finding was the significantly higher AMH expression in luteal cells during the luteal phase than during the follicular phase, suggesting that luteal AMH expression varies with reproductive stage. AMH expression in antral follicles was numerically higher in younger sows than in older sows, although this difference was not statistically significant, and no significant association was observed between AMH expression and ovulation number. These findings warrant further investigation into the physiological role of AMH in luteal cells. Because circulating AMH concentrations and longitudinal reproductive outcomes were not evaluated, the potential value of AMH for predicting reproductive longevity or guiding herd management remains uncertain. Future studies should clarify the relationship between ovarian AMH expression and circulating AMH concentrations and assess their associations with lifetime reproductive performance and sow longevity before considering practical applications in gilt selection, sow retention, or culling decisions.

## Figures and Tables

**Figure 1:**
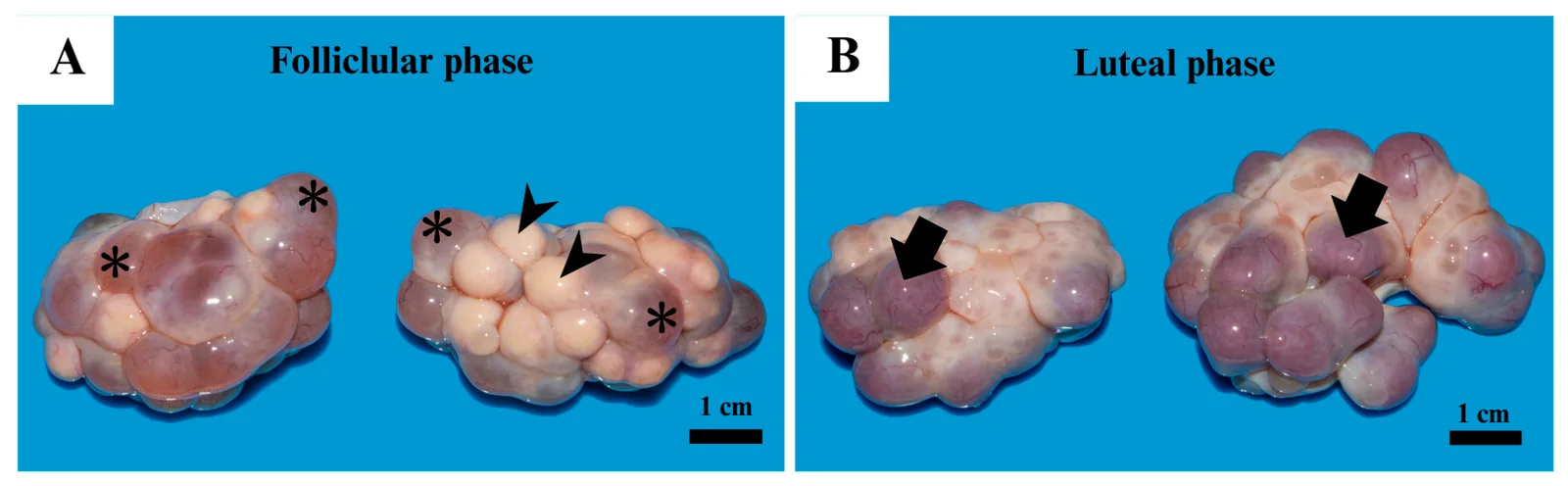
Reproductive phases in a hyperprolific sow. (**A**) Follicular phase showing multiple developing follicles at various stages, including a dominant follicle (asterisk) and a regressing corpus luteum (arrowhead). (**B**) Luteal phase characterized by the presence of post-ovulatory structures, particularly the corpus luteum (arrow), with no visible dominant follicle. Scale bar = 1 cm (bottom right of each image).

**Figure 2:**
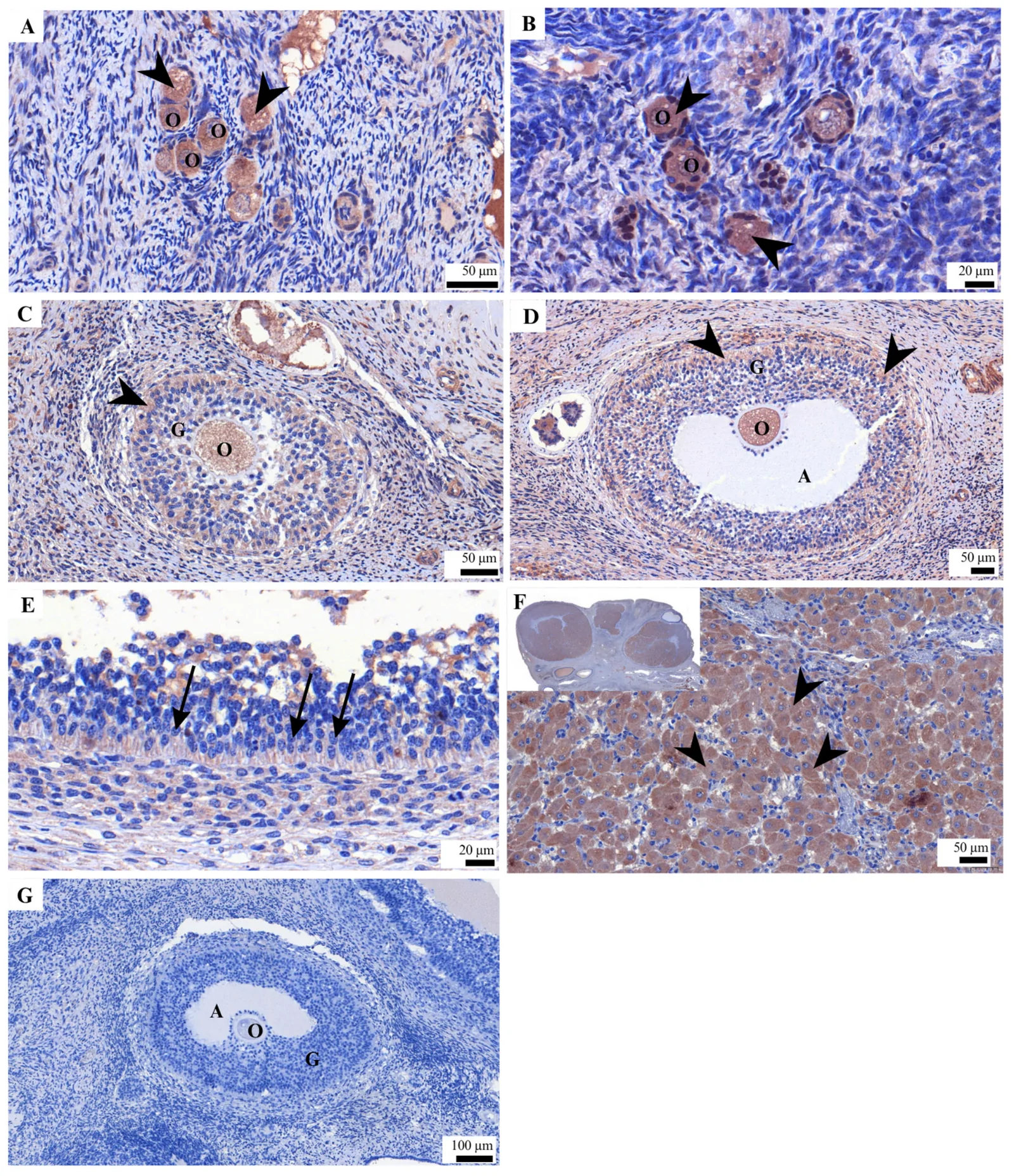
Immunoexpression of anti-Müllerian hormone (AMH) in ovarian follicles of a hyperprolific sow. (**A**) Primordial follicle; (**B**) primary follicle; (**C**) secondary follicle; and (**D**) antral follicle. (**E**) Immunoreactivity (arrow) is localized in the cytoplasm of granulosa cells with varying intensity. (**F**) Immunostaining in the corpus luteum showing AMH expression in the cytoplasm of luteal cells. (**G**) Negative control showing absence of brown staining. Scale bars are shown at the bottom right of each panel. (A, antrum; G, granulosa cell; O, oocyte). Arrowheads indicate AMH immunostaining.

**Table 1:** Average sow characteristics and reproductive performance parameters included in the study, presented as mean ± standard deviation (SD).

Variables	Mean ± SD	Range
Age at culling (d)	787.5 ± 325.6	463–1550
Parity 1 (*n* = 10)	472.6 ± 15.1	463–501
Parities 2–5 (*n* = 12)	728.2 ± 181.6	544–919
Parities 6–9 (*n* = 9)	1216.6 ± 125.2	1165–1550
Body weight at culling (kg)	215.7 ± 29.1	174.0–281.5
Parity 1 (*n* = 10)	207.7 ± 24.3	174.0–251.5
Parities 2–5 (*n* = 12)	210.7 ± 33.8	174.0–276.5
Parities 6–9 (*n* = 9)	230.3 ± 23.6	200.0–281.5
Parity number at culling	3.3 ± 2.3	1–9
Ovulation number (*n* = 26)	25.5 ± 7.9	10–40
Total number of piglets born per litter		
Parity 1 (*n* = 10)	15.9 ± 2.5	12–20
Parities 2–5 (*n* = 12)	16.9 ± 4.7	5–25
Parities 6–9 (*n* = 9)	17.9 ± 4.3	3–31

**Table 2:** Immunoexpression (H-score) of anti-Müllerian hormone (AMH) classified by parity group, ovulation rate class, and ovarian cycle stage (least-square means ± SEM).

Variables	Follicular Stage				Corpus Luteum
	Primordial	Primary	Secondary	Antral	
Number of observations	579	169	383	365	183
Means ± SD	98.6 ± 67.3	109.3 ± 73.3	73.1 ± 62.9	80.0 ± 51.7	92.8 ± 57.3
Parity numbers					
1	103.9 ± 34.2 ^a^	95.2 ± 35.7 ^a^	84.7 ± 25.5 ^a^	91.1 ± 20.3 ^a^	116.0 ± 22.9 ^a^
2–5	88.3 ± 24.9 ^a^	125.2 ± 32.3 ^a^	48.2 ± 20.5 ^a^	66.4 ± 15.6 ^a^	81.2 ± 18.5 ^a^
6–9	112.3 ± 25.1 ^a^	103.4 ± 28.6 ^a^	92.9 ± 23.2 ^a^	80.4 ± 16.4 ^a^	103.9 ± 19.6 ^a^
*p* value	0.687	0.520	0.298	0.156	0.853
Ovulation rate					
Low	90.4 ± 29.7 ^a^	108.5 ± 33.8 ^a^	75.4 ±24.3 ^a^	74.8 ±19.3 ^a,b^	96.5 ± 21.9 ^a^
Moderate	108.6 ± 24.0 ^a^	115.6 ± 28.6 ^a^	100.9 ± 19.9 ^a^	111.4 ± 14.1 ^a^	117.1 ± 16.8 ^a^
High	105.5 ± 29.9 ^a^	99.6 ± 33.9 ^a^	49.6 ± 25.4 ^a^	51.7 ± 19.3 ^b^	87.6 ± 22.8 ^a^
*p* value	0.656	0.803	0.504	0.749	0.606
Reproductive stage					
Follicular (*n* = 6)	87.4 ± 33.6 ^a^	112.3 ± 37.7 ^a^	65.2 ± 27.8 ^a^	68.9 ± 23.3 ^a^	24.7 ± 21.9 ^a^
Luteal (*n* = 31)	101.7 ± 13.8 ^a^	108.6 ± 15.9 ^a^	75.1 ± 12.9 ^a^	82.8 ± 10.4 ^a^	100.9 ± 10.8 ^b^
*p* value	0.529	0.994	0.292	0.254	<0.001

^a,b^ Within each column, values with different superscript letters differ significantly (*p* < 0.05). *p* values indicate the statistical significance of the overall effect of parity, ovulation rate class, or reproductive stage on AMH expression within each column, as determined by the corresponding GLM.

## Data Availability

The raw data supporting the conclusions of this article will be made available by the authors on request.
